# Reactivation of hepatitis B virus with mutated hepatitis B surface antigen in a liver transplant recipient receiving a graft from an antibody to hepatitis B surface antigen– and antibody to hepatitis B core antigen–positive donor

**DOI:** 10.1111/j.1537-2995.2011.03537.x

**Published:** 2012-09

**Authors:** Annette Blaich, Michael Manz, Alexis Dumoulin, Christian G Schüttler, Hans H Hirsch, Wolfram H Gerlich, Reno Frei

**Affiliations:** Division of Clinical Microbiology, University Hospital Basel; the Department of Gastroenterology, St Clara Hospital; and the Institute for Medical Microbiology, Department of Biomedicine, University of BaselBasel, Switzerland; and the Institute of Medical Virology, Justus-Liebig-University GiessenGiessen, Germany

## Abstract

**BACKGROUND:**

Fresh-frozen plasma (FFP) may contain antibodies to hepatitis B surface antigen (HBsAg, anti-HBs). These anti-HBs may lead to a misinterpretation of the actual hepatitis B immune status. Furthermore, they may not only confer protection against hepatitis B virus (HBV), but may also favor the selection of HBsAg mutants.

**CASE REPORT:**

We report a case of de novo HBV infection in a HBV-naïve recipient with alcoholic liver disease, who received a liver from a donor with antibodies to hepatitis B core antigen (HBcAg, anti-HBc) and anti-HBs.

**RESULTS:**

A lookback investigation revealed the following: 1) Due to anti-HBs passively acquired through FFP, the recipient was considered immune to HBV and did not receive anti-HBV prophylaxis. 2) Within 1 year after transplantation he developed hepatitis B in absence of any elevated liver enzymes after the anti-HBs by FFP declined. 3) Despite an infection with HBV-containing wild-type HBcAg, the patient did not seroconvert to anti-HBc positivity. 4) The replicating HBV encoded two HBsAg mutations, first sQ129R and 4 months later sP127S. They map to the highly conserved “α” determinant of the HBsAg loop.

**CONCLUSION:**

1) Passive transfer of anti-HBs from FFP led to an erroneous pretransplant diagnosis of HBV immunity when the patient was in fact HBV-naïve. 2) HBsAg mutations might have been selected in escape from donor's actively produced anti-HBs and the recipient's anti-HBs by FFP might have favored this selection. 3) It is doubtful whether hepatitis B immunoglobulin could have prevented the reactivation. 4) Antiviral prophylaxis would have been crucial.

The shortage of organs urges many transplantation centers to use marginally suitable grafts for orthotopic liver transplantation (OLT), for example, from donors with antibody to hepatitis B core antigen (anti-HBc), which is a marker of past hepatitis B virus (HBV) infection. However, transplanting such organs requires special caution. After a resolved HBV infection, the viral genome can persist as covalently closed circular DNA (cccDNA) in the liver. The expression and replication of these occult genomes is kept at a low level by the immune system, but the virus may reactivate in the graft during immunosuppressive therapy.^1^,^2^ HBV-naïve patients who receive such grafts are at high risk for developing active HBV infection.^2^,^3^ Guidelines of the European Association for the Study of the Liver (EASL) and the American Association for the Study of Liver Diseases (AASLD) strongly recommend antiviral prophylaxis in these patients. In recipients with a previous HBV infection (anti-HBc and antibody to hepatitis B surface antigen [anti-HBs] positive) who are at lowest risk for developing hepatitis B, the need for prophylaxis is controversial; in all other recipients it is mandatory. Vaccination before OLT lowers the risk.^2^,^3^ We describe a case of a HBV-naïve recipient who received a graft from an anti-HBc–positive donor. Because of the confounding serologic situation before transplantation, he did not receive anti-HBV prophylaxis and developed a de novo HBV infection. We identify the factors that contributed to erroneous omission of HBV prophylaxis, describe the course and treatment of the reactivated HBV infection, characterize the patient's HBV variants, and propose measures for prevention. We have received the patient's consent to publish information concerning his case.

## CASE REPORT

In August 2006, a 52-year-old man suffering from alcoholic liver cirrhosis (Child-Pugh Score B) presented with upper gastrointestinal bleeding from esophageal varices. After alcohol misuse for many years, he had been abstinent since February 2006. No risk factors for hepatitis B were known. Over the following 3 days he received solvent/detergent-treated (S/D) fresh-frozen plasma (FFP) (Octaplas, Octopharma, Lachen, Switzerland) and 10 red blood cell (RBC) units. Serum taken on Day 4 contained total antibodies to hepatitis A virus (HAV, anti-HAV) and a low level of 28.1 IU/L anti-HBs; all other HBV markers were negative (Table 1, 8/19/2006). Histologic examination of a liver biopsy showed cirrhosis with active, chronic alcoholic hepatitis. Staining for intracytoplasmatic hepatitis B surface antigen (HBsAg) was negative.

**TABLE 1:** Course of hepatitis B and A serology, HBV viral load, and liver enzymes

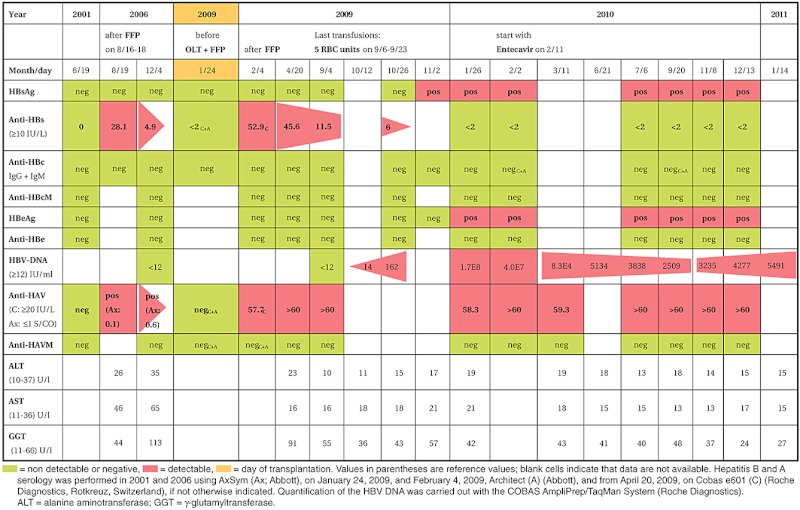

In December 2006, HBV markers including HBV DNA were negative, anti-HBs was at 4.9 IU/L, immunoglobulin M to HAV (anti-HAVM) was not detected, but anti-HAV was still present (Table 1, 12/4/2006) and antibody to hepatitis D virus was negative (data not shown). In June 2007 the patient was waitlisted for liver transplantation. Until November 2007 he needed occasional paracentesis and was treated with diuretics. In December 2007 his condition deteriorated and he needed large-volume paracentesis weekly. His Mayo End-Stage Liver Disease (MELD) score increased from 14 to 17 points.

On January 24, 2009, he received a liver graft from a 47-year-old deceased donor. At the time of transplantation the donor was HBsAg negative, anti-HBc positive, and had 40 IU/L anti-HBs. He had immunoglobulin G to HAV (anti-HAVG) but no anti-HAVM. HBV DNA was not detectable in the donor's serum at a detection limit of 12 IU/mL. Histology of the graft revealed mild macrosteatosis (approx. 20%) and focal perisinusoidal fibrosis without evidence of active hepatitis or septal fibrosis. The findings suggested past HBV and HAV infection. The recipient did not have HBsAg, anti-HBc, anti-HBs, anti-HAVG, or anti-HAVM just before OLT (Table 1, 1/24/2009). During and immediately after transplantation the patient received 13 units of FFP, seven granulocyte concentrates, and 2 RBC units; in September 2009, the patient received 5 RBC units; and thereafter no blood products were administered. After transplantation, the patient followed an immunosuppressive regimen with prednisone, tacrolimus, and mycophenolate mofetil. He received no HBV prophylaxis and 11 days after transplantation he had 52.9 IU/L anti-HBs. Furthermore, he had 57.7 IU/L anti-HAVG (Table 1, 2/4/2009). Retrospectively, in September 2010, we reviewed the serum samples taken before and after transplantation (Table 1, 1/24/2009 and 2/4/2009), confirmed the anti-HBs, anti-HAVG, and anti-HAVM results on a second test platform, and got notice of a negative HBV and HAV serology from 2001 (Table 1, 6/19/2001). In May 2009, prednisone was discontinued. The transaminases were monitored at irregular intervals between 1 and 4 months and were in the normal range. HBV markers were initially not determined after OLT.

One year after transplantation (Table 1, 1/26/2010 and 2/2/2010), the patient's serum was tested in context of the Swiss Transplant Cohort Study, according to schedule. Surprisingly, HBsAg and hepatitis B e antigen (HBeAg) were highly positive, but anti-HBc was negative and remained negative throughout the entire observation period. Quantitative HBV DNA polymerase chain reaction (PCR) yielded a highly replicative HBV infection with more than 10^8^ IU/mL whereas the liver enzymes were still normal and remained normal.

Therapy with entecavir was initiated in February 2010. Within 4 weeks the serum HBV DNA decreased to values of less than 10^5^ IU/mL, but thereafter it remained between 10^3^ and 10^4^ IU/mL. HBsAg in the sample taken on February 2, 2010, showed a very high value of 173,000 IU/mL (determined quantitatively on Architect, Abbott Laboratories, Wiesbaden, Germany) and even increased significantly to 284,000 IU/mL on June 21, 2010. After the follow-up on January 14, 2011, the daily dose of 0.5 mg of entecavir was increased to 1 mg. This was followed by a transient decrease of HBV DNA to 1632 IU/mL on February 28, 2011. However, at the last follow-up on September 19, 2011, the viral load was at 2898 IU/mL. Switching to tenofovir was considered. To date, the patient has been in good health on antiviral and immunosuppressive treatment with entecavir and tacrolimus-mycophenolate mofetil.

## RESULTS

### A lookback investigation was initiated

We found that the decline of anti-HBs after transplantation to values of less than 10 IU/L was mirrored by the increase of HBV DNA and HBsAg in the recipient (Table 1, 4/20/2009-11/2/2009). Furthermore, it was shown that the PCR for HBV DNA and immunostaining for HBsAg, HBcAg, and hepatitis delta virus antigen were negative in the recipient's pretransplant paraffin-embedded liver biopsy from August 2006. A HBV DNA PCR procedure from the paraffin-embedded donor's biopsy from January 2009 was negative as well. Detection of HBV cccDNA by PCR in liver tissues from anti-HBs– and anti-HBc–positive persons is only promising from freshly taken biopsies and was therefore not performed. The potential effect of passively administered anti-HAV and anti-HBs was now considered: The patient had received 200 mL of S/D FFP in August 2006 containing 727 IU/L anti-HBs and 2000 IU/L anti-HAV and 400 mL of S/D FFP containing 479 IU/L anti-HBs and 3000 IU/L anti-HAV.

**Fig. 1:**
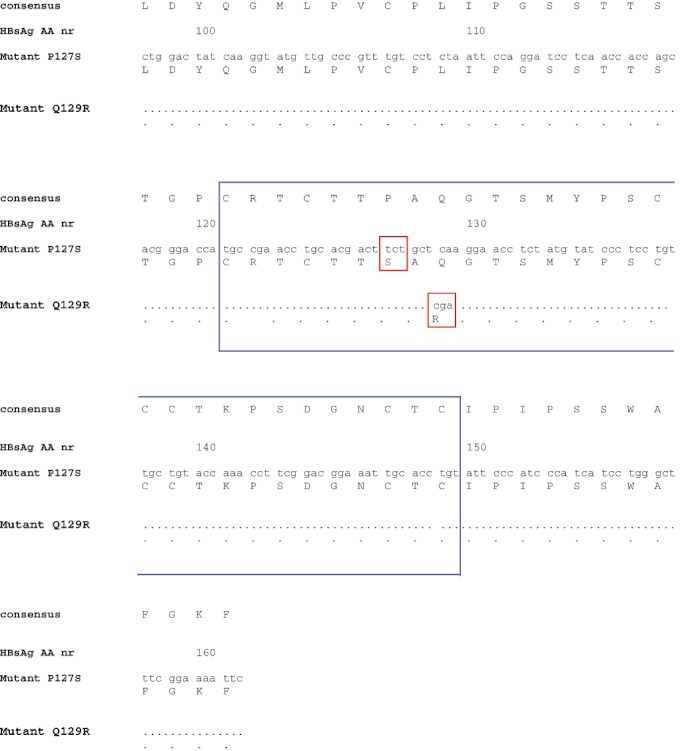
Nucleotide and deduced amino acid (AA) sequences of the HBsAg loop of the HBV mutants P127S and Q129R and the wild-type–HBsAg Genotype D consensus AA sequence derived from GenBank Accession Numbers AY721605, M23138, and X02496. Sequences of the “a” determinant (AA 121-149) are within the large rectangle, and the mutations are highlighted by small rectangles

The HBV strain was characterized in the blood sample taken on February 2, 2010, just before entecavir therapy. Direct sequencing of a PCR product covering the entire surface gene (as described in Bremer et al.^4^) showed HBV Genotype D, HBsAg Subtype ayw2. In addition, the complete genome was amplified and cloned to analyze the heterogeneity of the viral quasispecies. Twelve clones were isolated and sequenced. The core gene showed no relevant mutations. The reverse transcriptase domain of the HBV DNA polymerase contained polymorphic exchanges rtA21S and rtQ130P, but no resistance-associated mutations. However, the “a” determinant of the major HBsAg protein and the main target for neutralizing anti-HBs showed in 11 clones a mutation at position Q129 to R, except in one clone, which had a mutation at position P127 to S (Fig. S1, available as supporting information in the online version of this paper; the sequences of the mutated HBsAg loops are displayed in Fig. 1). In the plasma sample taken on June 21, 2010, after 4 months of entecavir therapy, the mutant Q129R was no longer detected, but P127S predominated. The reverse transcriptase domain was unaltered.

The blood products for the OLT and the 5 RBC units in September 2009 were supplied from blood donation centers, which screen all donations for HBsAg and since July 2, 2007, for HBV DNA as well. This screening is performed on every single donation without pooling. A lookback investigation revealed that all of the five donors for the 5 RBC units were anti-HBc negative at their latest donation in 2010 or 2011. Two of the five donors had anti-HBs of more than 500 IU/L and three of five were anti-HBs negative (Table S1, available as supporting information in the online version of this paper).

## DISCUSSION

We report the case of a HBV reactivation with HBsAg mutant virus after transplantation of an organ from an anti-HBs– and anti-HBc–positive donor. Based on the results of lookback investigations, it is extremely unlikely that the blood products that were administrated during and after the OLT were the source of the HBV infection. Considering the time course of the infection, a transmission through other sources before the OLT is also very unlikely. Furthermore, a HBV infection superimposed on an end-stage alcoholic liver disease would have led to a serious deterioration in our patient before the OLT. Therefore, the most probable source for the HBV infection in our recipient is the infected graft of the anti-HBc–positive donor.

Due to an anti-HBs level of more than 10 IU/L and positive anti-HAV (August 2006) the patient was erroneously considered immune to HBV and HAV. The (serologic) vaccination status was not reviewed on the first visit to the transplant center (March 2007), or the status was reevaluated when the patient was waitlisted for transplantation (June 2007), in contradiction of the guidelines.^5^ The consequence was that this patient with life-threatening chronic liver disease (CLD) was not vaccinated against HAV and HBV although in retrospect he was not immune and did not receive HBV prophylaxis after OLT.

Physicians should be aware of this pitfall. The quantity, origin, and specificity of relevant antibodies should be assessed in all transplantation candidates. Not only prophylactic or therapeutic immunoglobulin preparations, but also FFP and plasma-containing products such as RBC or thrombocyte concentrates, may cause misleading serologic results in the recipient. In contrast to active immunity, passively acquired antibodies slowly disappear. Therefore, antibodies should be assayed repeatedly, and the results should be interpreted together with the transfusion history of the patient to ensure that they persist and represent true active immunity. Other pitfalls in serology include cross-reacting antibodies, nonspecific reactions, and polyclonal B-cell stimulation, which may mimic specifically induced antibodies. Therefore, the results should be checked by a second method. Ideally, a patient's status should be assessed long before transplantation, to vaccinate patients with nonviral CLD.

To date, there is no standardized HBV prophylaxis regimen in the transplantation setting. Hepatitis A or B in patients with CLD and in adults aged 40 years or more is associated with a more severe clinical course and higher mortality.^6^ Guidelines recommend vaccinating patients with CLD and candidates for OLT against HAV, HBV, and other vaccine-preventable agents as early as possible.^5^,^7^,^8^ HBV and HAV vaccination is less effective in patients with advanced disease and after transplantation.^6^,^9^ Serial anti-HBs should be assessed before and after transplantation to recognize hypo- and nonresponders and if necessary to vaccinate repeatedly and with higher doses.^9^ Protective anti-HBs titers of greater than 100 IU/L are necessary to minimize breakthrough infections and emergence of escape mutants.^1^,^10^

Optimal antiviral prophylaxis after OLT in recipients of grafts from anti-HBc–positive donors is the subject of debate.^11^ Early treatment with lamivudine before the appearance of HBV DNA in serum is recommended.^12^ The experience in our case is that a cure cannot be achieved when the graft is already heavily infected after reactivation.

During and after transplantation the patient received FFP from single Swiss donors. The anti-HBs content in the FFP is not known, but the appearance of anti-HBs after OLT showed that at least one of the donors was anti-HBs positive. Since anti-HBc was not transferred by FFP at detectable levels, it is probable that the anti-HBs in the FFP were induced by vaccination. The prevalence of anti-HBc in the Swiss donor population has been reported as less than 2%.^13^ The passively acquired anti-HBs were probably able to delay reactivation until 9 months after transplantation, but could not prevent it, because they disappeared. Our data suggest that in such cases, both anti-HBs and HBsAg should be monitored for at least 1 year after transplantation. Furthermore, the anti-HBs may have favored the selection of HBsAg mutants. Therefore, it is very doubtful whether hepatitis B immunoglobulin alone could have prevented a reactivation on long-term view. Mutants at Positions 127 (e.g., P127S) and 129 have been described in the context of escape from hepatitis B immunoglobulin and wild-type and vaccine-induced anti-HBs, Q129R only in escape from the latter.^14^,^15^ Alternatively, one or both mutants may have already been selected by the donor's immune system before transplantation; anti-HBs was well detectable in the donor. Probably, the first mutant (Q129R) was transmitted with the graft and the anti-HBs from the FFP might have favored its selection for a while. With the decline of the anti-HBs, a possibly fitter variant (P127S) emerged. In wild-type HBV of Genotype D, both 127P and T may occur. 127S is functionally similar to 127T but may still cause an altered HBs antigenicity favoring escape from a low level of anti-HBs.

This case also demonstrates that it is very important to recognize occult HBV infection in a liver donor. After exposure to HBV, anti-HBc usually persists irrespective of ongoing hepatitis or resolution.^16^ Anti-HBc could have been useful for identifying the risk of HBV reactivation in the donor liver. Unfortunately, approximately 15% of subjects with occult HBV DNA in the liver do not have anti-HBc.^16^ Anti-HBs only is a marker for vaccination in the past, for passively acquired antibodies^17^ or rarely a sign of resolved or occult HBV infection.^1^,^18^

HBcAg is the most immunogenic HBV component,^19^ but it sometimes fails to induce anti-HBc as in our patient. The following mechanisms may underlie the profile of an anti-HBc–negative HBV infection: 1) Studies in the woodchuck model showed that infection with very low doses may lead to primary occult infections which result in T cell–mediated protection but no detectable antibody formation.^20^ Although the amount of HBV may have been low, this mechanism is probably not present here because there is no resolution of the infection and no hint for protective T-cell immunity in the recipient. 2) Mutations within the precore-core gene may lead to synthesis of a truncated HBcAg that does not induce anti-HBc or induces it only at a low level, but these mutations are rare^19^,^21^ and were absent in our patient. 3) Low-sensitivity serologic assays may fail to detect anti-HBc. In the serum of our patient, anti-HBc was clearly negative on two different days on two various platforms (Table 1, 2/2/2010 and 9/20/2010). 4) The lack of anti-HBc production might be due to a generalized antibody deficiency (i.e., hypo- or agammaglobulinemia). However, the patient repeatedly had inconspicuous gammaglobulin fractions in serum protein electrophoresis and well-detectable antibodies against Epstein-Barr virus and varicella zoster virus. 5) Immunosuppression at the time of acquiring a new infection may impair persistently the capability to develop immunity to this new agent. Failure to produce anti-HBc after new HBV infections has mostly been described in immunocompromised patients, that is, in human immunodeficiency virus infection, in the context of transplantation,^19^,^21^ and after immunosuppressive cancer therapy.^22^ The effect of immunosuppression seemed to act stronger on HBV than on other pathogens in the recipient, because he was able to develop antibodies to transplantation-associated primary cytomegalovirus infection (unpublished data). 6) Theoretically, anti-HBc may have been produced but bound by an excess of free HBcAg. However, previous work^22^ had shown that chronically HBV-infected patients with high viremia and without anti-HBc had no free HBcAg in the serum.

The clinical course of the reactivated HBV infection in the graft was surprisingly mild in spite of very strong replication. HBV reinfection of an originally HBV-negative graft in a HBV-infected recipient often causes rapid deterioration of the liver. Such recipients have experienced severe immunopathogenesis before transplantation and have obviously retained enough cytotoxic memory cells to retain the potential for immunopathogenesis when reinfection occurs. Obviously, the absence of any active immune reaction against HBV antigens in the patient described here favors a benign course.

In view of no detectable liver damage before and during entecavir therapy one could ask whether this therapy is necessary. It has only been partially successful and leaves viremia at relatively high levels of approximately 5000 IU/mL. The already very high HBsAg concentration in serum increased significantly during the therapy. This suggests that HBV continues to spread within the liver. Antiviral treatment blocks reverse transcription, genome maturation, and, thus, generation of new cccDNA, but it cannot diminish production of viral antigens. The high amount of HBsAg suggests that the liver contained a very high load of cccDNA which was fully expressed at the level of transcription and translation. The limited effect of the antiviral therapy is obviously the consequence of a very high production of RNA pregenomes, DNA polymerase, and immature core particles, which finally results in a relatively large amount of secreted and infectious HBV particles. Currently, the only recognizable benefit of entecavir in this case is the reduction of infectivity of the blood to levels not dangerous for normal household contact.

We are convinced that the transient anti-HAV in 2006 and anti-HBs in 2006 and 2009 were passively transferred by FFP: anti-HAV and anti-HBs were absent in 2001 and shortly before transplantation. The antibody content in the FFP in 2006 was high: anti-HAV and anti-HBs was immediately detectable after administration of FFP in 2006 and 2009 and anti-HBs steadily declined thereafter. Anti-HBs have no longer been detectable since January 2010. Surprisingly, anti-HAVG did not decline after transplantation (Table 1). There is no evidence for the patient having an HAV infection shortly before or after the transplantation. The reason for the persistent anti-HAV is not clear; one hypothesis may be that the antibodies come from adoptive immunity provided by B cells present in the graft. Transfer of adoptive immunity has been described for anti-HBs–positive liver donors. With timely HBV antiviral therapy after transplantation, the donor's HBV humoral immunity could possibly have been established in the recipient as well and could have controlled HBV later on.^12^

In summary, humoral immunity against microbial agents should be interpreted with caution after administration of FFP or blood products. To ensure active immunity, protective antibody levels should be assessed repeatedly over time. Patients with CLD should be immunized early against preventable diseases. Early prophylaxis should be given if the liver graft may contain occult HBV. Anti-HBc is not a reliable screening marker for ongoing HBV infection in immunocompromised patients.

## Abbreviations

anti-HAVG =: immunoglobulin G to hepatitis A virus
anti-HAVM =: immunoglobulin M to hepatitis A virus
cccDNA =: covalently closed circular DNA
CLD =: chronic liver disease
OLT =: orthotopic liver transplantation

## SUPPORTING INFORMATION

**Fig. S1.** Two-dimensional model showing the mutations P127S and Q129R in the HBsAg loop.

**Table S1.** HBV status of the 5 donors for the 5 RBC units administrated to our liver transplant recipient in September 2009.

## References

[1] Gerlich WH, Bremer C, Saniewski M, Schüttler CG, Wend UC, Willems WR, Glebe D (2010). Occult hepatitis B virus infection: detection and significance. Dig Dis.

[2] Cholongitas E, Papatheodoridis GV, Burroughs AK (2010). Liver grafts from anti-hepatitis B core positive donors: a systematic review. J Hepatol.

[3] Avelino-Silvo VI, D'Albuquerque LA, Bonazzi PR, Song AT, Miraglia JL, de Brito Neves A, Abdala E (2010). Liver transplant from anti-HBc-positive, HBsAg-negative donor into HBsAg-negative recipient: is it safe? A systematic review of the literature. Clin Transplant.

[4] Bremer CM, Saniewski M, Wend UC, Torres P, Lelie PN, Gerlich WH, Glebe D (2009). Transient occult hepatitis B virus infection in a blood donor with high viremia. Transfusion.

[5] Danzinger-Isakov L, Kumar D, the AST Infectious Diseases Community of Practice (2009). Guidelines for vaccination of solid organ transplant candidates and recipients. Am J Transplant.

[6] Keeffe EB (2005). Acute hepatitis A and B in patients with chronic liver disease: prevention through vaccination. Am J Med.

[7] Fiore AE, Wasley A, Bell BP, Advisory Committee on Immunization Practices (ACIP) (2006). Prevention of hepatitis A through active or passive immunization: recommendation of the Advisory Committee on Immunization Practices. MMWR Recomm Rep.

[8] Mast EE, Weinbaum CM, Fiore AE, Alter MJ, Bell BP, Finelli L, Rodewald LE, Douglas JM, Janssen RS, Ward JW, Advisory Committee on Immunization Practices (ACIP) Centers for Disease Control and Prevention (CDC) (2006). A comprehensive immunization strategy to eliminate transmission of hepatitis B virus infection in the United States: recommendations of the Advisory Committee on Immunization Practices, Part II: immunization of adults. MMWR Recomm Rep.

[9] Keeffe EB (2006). Hepatitis A and B superimposed on chronic liver disease: vaccine-preventable diseases. Trans Am Clin Climatol Assoc.

[10] Bauer T, Günther M, Bienzle U, Neuhaus R, Jilg W (2007). Vaccination against hepatitis B in liver transplant recipients: pilot analysis of cellular immune response shows evidence of HBsAg-specific regulatory T cells. Liver Transpl.

[11] Saab S, Waterman B, Chi AC, Tong MJ (2010). Comparison of different immunoprophylaxis regimens after liver transplantation with hepatitis B core antibody-positive donors: a systematic review. Liver Transpl.

[12] Shouval D (2007). Adoptive transfer of immunity to HBV in liver transplant patients: a step forward toward the proof of concept for therapeutic vaccination or a transient immunologic phenomen?. Liver Transpl.

[13] Niederhauser C, Taleghani BM, Graziani M, Stolz M, Tinguely C, Schneider P (2008). Blood donor screening: how to decrease the risk of transfusion-transmitted hepatitis B virus?. Swiss Med Wkly.

[14] Mele A, Tancredi F, Romanò L, Giuseppone A, Colucci M, Sangiuolo A, Lecce R, Adamo B, Tosti ME, Taliani G, Zanetti AR (2001). Effectiveness of hepatitis B vaccination in babies born to hepatitis B surface antigen-positive mothers in Italy. J Infect Dis.

[15] Avellón A, Echevarria JM (2006). Frequency of hepatitis B virus “a” determinant variants in unselected Spanish chronic carriers. J Med Virol.

[16] Raimondo G, Navarra G, Mondello S, Costantino L, Colloredo G, Cucinotta E, Di Vita G, Scisca C, Squadrito G, Pollicino T (2008). Occult hepatitis B virus in liver tissue of individuals without hepatic disease. J Hepatol.

[17] Lim YA, Hyun BH, Kim DY (2002). Effect of transfusion of fresh-frozen plasma on recipient's antibodies to hepatitis B surface antigen and hepatitis B surface antigen status in countries where hepatitis B is endemic. Vox Sang.

[18] Torbenson M, Thomas DL (2002). Occult hepatitis B. Lancet Infect Dis.

[19] Kantelhardt VC, Schwarz A, Wend U, Schüttler CG, Willems WR, Trimoulet P, Fleury H, Gerlich WH, Kann M (2009). Re-evaluation of anti-HBc non-reactive serum samples from patients with persistent hepatitis B infection by immune precipitation with labelled HBV core antigen. J Clin Virol.

[20] Gujar SA, Michalak TI (2009). Primary occult hepadnavirus infection induces virus-specific T-cell and aberrant cytokine responses in the absence of antiviral antibody reactivity in the woodchuck model of hepatitis B virus infection. J Virol.

[21] Avettand-Fenoel V, Thantibodyut D, Katlama C, Poynard T, Thibault V (2006). Immune suppression as the etiology of failure to detect anti-HBc antibodies in patients with chronic hepatitis B virus infection. J Clin Microbiol.

[22] Possehl C, Repp R, Heermann KH, Korec E, Uy A, Gerlich WH, DeBac C, Gerlich WH, Taliani G (1992). Chronically evolving viral hepatitis (Archives of virology. Supplementa. Absence of free core antigen in anti-HBc negative viremic hepatitis B carriers.

