# Supplementary material for: Reactivation of hepatitis B virus with mutated hepatitis B surface antigen in a liver transplant recipient receiving a graft from an antibody to hepatitis B surface antigen– and antibody to hepatitis B core antigen–positive donor
Source: Transfusion. 2012 Sep;52(9):1999–2006. doi: 10.1111/j.1537-2995.2011.03537.x (PMC3465803; doi:10.1111/j.1537-2995.2011.03537.x)
Supplement: Supplementary file 1 [file trf0052-1999-SD1.doc]

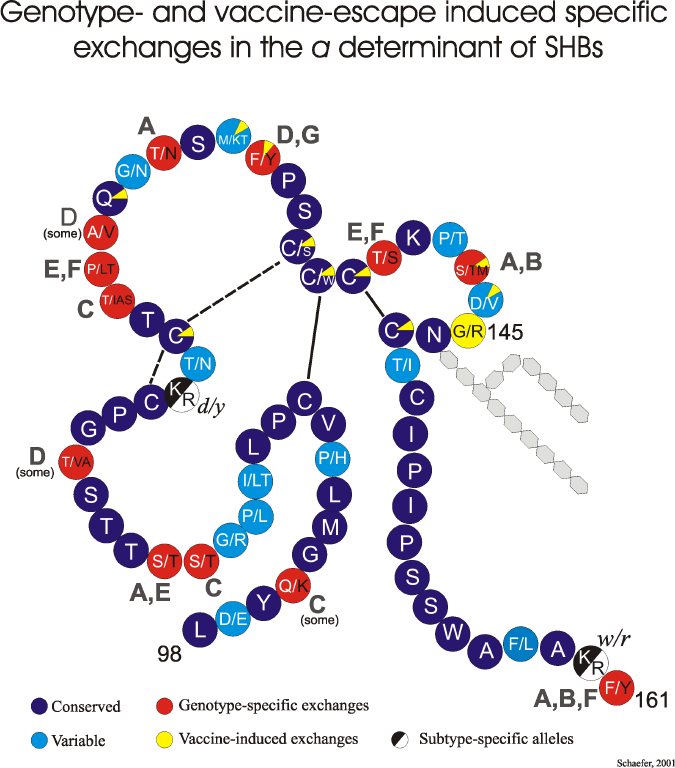


**"α" determinant**

**P127S**

**Q129R**

**Mutations**

**Figure S1:** Mutations P127S and Q129R in the HBsAg loop. The topology of the two-dimensional model and the cross-linking by disulfide bonds is speculative. The amino acids (AA) are stated in the one-letter code from position 98 to 161. The "α" determinant comprises the AA 121 to 149. Dark blue = conserved positions; light blue = variable; red = genotype specifies by the capital letters. Adapted from reference 1 with permission from S. Karger AG, Basel, Switzerland.
