# Supplementary material for: Reactivation of hepatitis B virus with mutated hepatitis B surface antigen in a liver transplant recipient receiving a graft from an antibody to hepatitis B surface antigen– and antibody to hepatitis B core antigen–positive donor
Source: Transfusion. 2012 Sep;52(9):1999–2006. doi: 10.1111/j.1537-2995.2011.03537.x (PMC3465803; doi:10.1111/j.1537-2995.2011.03537.x)
Supplement: Supplementary file 2 [file trf0052-1999-SD2.doc]

|  |  |  |  |  |  |
| --- | --- | --- | --- | --- | --- |
| **Donor** | **Donation date** | **Transfusion date** | **At donation** | |  |
|  |  |  | **HBsAg** | **HBV NAT** |  |
| Donor 1 | 8/25/2009 | 9/6/2009 | negative | negative |  |
| Donor 2 | 9/2/2009 | 9/10/2009 | negative | negative |  |
| Donor 3 | 9/2/2009 | 9/10/2009 | negative | negative |  |
| Donor 4 | 9/17/2009 | 9/23/2009 | negative | negative |  |
| Donor 5 | 9/17/2009 | 9/23/2009 | negative | negative |  |
|  |  |  |  |  |  |
|  |  |  | **At the latest donation** | |  |
| **Donor** | **Latest donation date** | **HBsAg** | **HBV NAT** | **Anti-HBc** | **Anti-HBs** |
|  |  |  |  |  |  |
| Donor 1 | 8/30/2011 | negative | negative | negative | negative |
| Donor 2 | 1/27/2010 | negative | negative | negative | 883.37 IU/l |
| Donor 3 | 11/20/2010 | negative | negative | negative | negative |
| Donor 4 | 3/21/2011 | negative | negative | negative | negative |
| Donor 5 | 7/20/2011 | negative | negative | negative | >1000 IU/l |
|  |  |  |  |  |  |

**Table S1:** HBV status of the 5 donors for the 5 RBC units administrated to our liver transplant recipient in September 2009.
 NAT*=*Nucleic acid testing.
